# Emergency Situation Prediction Mechanism: A Novel Approach for Intelligent Transportation System Using Vehicular Ad Hoc Networks

**DOI:** 10.1155/2015/218379

**Published:** 2015-05-10

**Authors:** P. Ganeshkumar, P. Gokulakrishnan

**Affiliations:** ^1^Department of Information Technology, PSNA College of Engineering and Technology, Dindigul, Tamil Nadu 624622, India; ^2^Department of Computer Science and Engineering, PSNA College of Engineering and Technology, Dindigul, Tamil Nadu 624622, India

## Abstract

In Indian four-lane express highway, millions of vehicles are travelling every day. Accidents are unfortunate and frequently occurring in these highways causing deaths, increase in death toll, and damage to infrastructure. A mechanism is required to avoid such road accidents at the maximum to reduce the death toll. An Emergency Situation Prediction Mechanism, a novel and proactive approach, is proposed in this paper for achieving the best of Intelligent Transportation System using Vehicular Ad Hoc Network. ESPM intends to predict the possibility of occurrence of an accident in an Indian four-lane express highway. In ESPM, the emergency situation prediction is done by the Road Side Unit based on (i) the Status Report sent by the vehicles in the range of RSU and (ii) the road traffic flow analysis done by the RSU. Once the emergency situation or accident is predicted in advance, an Emergency Warning Message is constructed and disseminated to all vehicles in the area of RSU to alert and prevent the vehicles from accidents. ESPM performs well in emergency situation prediction in advance to the occurrence of an accident. ESPM predicts the emergency situation within 0.20 seconds which is comparatively less than the statistical value. The prediction accuracy of ESPM against vehicle density is found better in different traffic scenarios.

## 1. Introduction

This work is motivated by the statistics of increased highway accidents and rise in death toll every day. In India, 1, 37,423 people were killed and 4, 69,900 people were injured in road traffic crashes in 2013 (NCRB 2013). Traffic fatalities increased by about 54.3 percent during the period 2003–2013 [1]. The World Health Organization (WHO) reports say that the road accident is the fifth cause of death in the world. Nearly 1.3 million people die in road accidents every year. The accident injury rate is 50 times greater than the death rate [2]. The total number of vehicles all over the world is estimated to be one billion in 2010 [3]. In 2013, the total number of vehicles is estimated to be 1.2 billion which leads to an increase of accidents and deaths [4].

Road traffic accidents have a high impact on the economy of the developing countries and affect the development of those countries [5]. Around 20% of Gross Domestic Product (GDP) of Indian economy suffers because of road accidents [6]. Statistics say that if the driver of the vehicle is provided emergency warning at least one-half second before an accident then 60% of highway accidents could be avoided [7].

**Figure 1:**
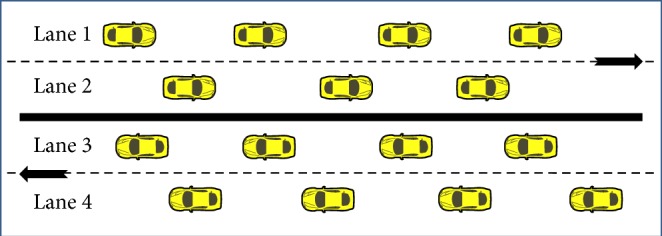
Indian four-lane express highway.

The objective of this paper is to design a framework to implement Intelligent Transportation System (ITS) using Vehicular Ad Hoc Network (VANET) with an intention to reduce the number of road accidents and to decrease the death toll caused by road accidents. An Emergency Situation Prediction Mechanism (ESPM), a proactive framework for accident prediction and prevention, is proposed to meet the objective. ESPM is designed for a four-lane express highway of Indian traffic system. In the existing Indian four-lane express highway the vehicles move in two lanes in left-hand side in one direction against the two lanes of the other side as shown in Figure 1. Four-lane express highway in India has a physical median or divider and generally has a speed limit of 60 km/h to 90 km/h [8].

ESPM attempts to predict the possibility of occurrence of an accident in advance before it occurs. An accident is said as an emergency situation in this paper. The Road Side Unit (RSU), the vehicles in the range of the RSU, and the four-lane express highway are considered in ESPM for emergency situation prediction and prevention.

Once the vehicles enter into the range of RSU, periodically the vehicles send a Status Report (SR) to RSU simultaneously the RSU performs traffic flow (TF) analysis. A Four-Lane Sensor Grid (FLSG) is designed to support ESPM for traffic flow analysis. ESPM performs prediction based on both SR and TF values. As the prediction process involves both the SR and TF values, the prediction accuracy (PA) is promisingly increased.

The highway road accidents can be categorized as head-on accidents, lateral impact, and rear-end accidents [9]. Sudden acceleration or deceleration of the vehicle is a major reason for rear-end accidents (i.e., chain of accidents) and head-on accidents. Improper or abnormal lane change in highway might cause lateral impact accidents. ESPM performs prediction for rear-end accidents, head-on accidents, and lateral impact accidents as discussed in Sections 5.3.1 and 5.3.2.

The rest of the paper is organized as follows. In Section 2, background of ESPM is presented.  Section 3 presents related systems.  Section 4 gives an overview of ESPM.  Section 5 presents detailed design of ESPM. Simulation setup and result evaluation are discussed in Sections 6 and 7, respectively. Finally, Section 8 concludes the ESPM framework.

## 2. Background

Intelligent Transportation System (ITS) is an emerging area of research over the past two decades. Transportation system becomes intelligent with emerging computing hardware, positioning system, sensor technologies, high speed telecommunication, and data processing.

ITS makes the travels of ease and comfort, it also provides reliable and improved travel safety. The environment gets benefit out of ITS because of low carbon emissions and air pollutions. Due to improvement in traffic efficiency and reduction of accident rate the economy of countries is improved [5, 10].

**Figure 2:**
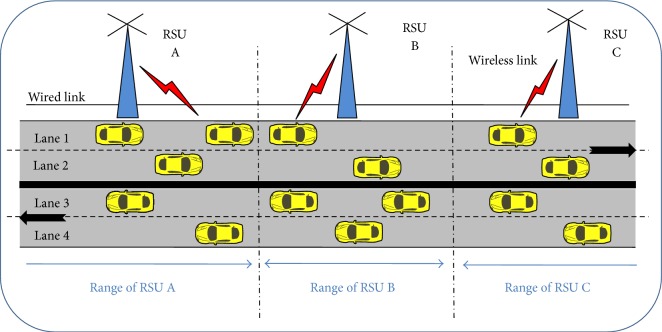
VANET Architecture.

VANET is a technology that uses moving vehicles as nodes and forms a Mobile Network. Vehicles communicate with each other via Vehicle to Vehicle communication (V2V) and with roadside base stations via Vehicle to Infrastructure communication (V2I) [11]. Vehicle to Vehicle communication is performed using Dedicated Short Range Communication (DSRC) and Vehicle to Roadside communication is performed using IEEE 802.11p [12] as shown in Figure 2.

ITS in VANET leads to the concept of Automated Highway System (AHS). AHS provides Safer Highway Transportation by making a vehicle to predict the actions of neighboring vehicles [13]. Establishment and maintenance of communication paths are more difficult in VANET due to the high mobility of the network. In order to prevent accidents, short reaction times from both system and driver are essential due to the high speed of the vehicles [14, 15]. Perception, modeling, decision making, and control are tough in real time safety analysis involving driver, vehicle, and the traffic environment itself [16].

## 3. Related Work

Caliendo et al. [17] considered vehicle density, traffic data, road conditions and weather for crash prediction in multilane roads. The authors have analyzed and classified severe accidents from total accidents. They have performed mathematical analyzes and provided a crash-prediction model for multilane roads. They have not considered important parameters such as vehicles and human (driver) behavior.

Hu et al. [18] have developed a traffic accident prediction scheme based on tracking of vehicles. Fuzzy self-organizing neural network method has been used for learning vehicle activity patterns. The authors have established a probability model for predicting traffic accidents. This model is suitable for urban vehicular scenario.

Sörstedt et al. [19] focus on the behavior or influence of the driver rather than vehicle and traffic conditions. A model with postulates is developed based on the behavior, actions, and influence of the driver on the vehicle. Other important parameters such as vehicle actions and road traffic conditions have not been considered.

Worrall et al. [20] propose a model named Intelligent Systems for Risk Assessment (ISRA). This model is developed with an aim to eliminate accidents by detecting risks and alerting other vehicles to take actions appropriately. Using artificial intelligence, this model attempts to assess the risk based on the dynamic behavior of the vehicle, environmental conditions, and ability of the human or driver. Risk assessment is made only based on less number of complex scenarios.

De Oña and Garach [21] used the Interactive Highway Safety Design Model (IHSDM) for analyzing the relationship between speed reduction and accidents in Spanish two-lane rural highways. IHSDM was actually developed for American two-lane rural highways. This mathematical model analyses the cause of accidents in tangent and curves of roads due to speed reduction. All scenarios of the rural highways were not considered.

Garcia-Lozano et al. [22] propose a warning service to the drivers in order to prevent from accidents due to dangerous road conditions and accidents caused by other vehicles. A part of the warning service, an alert message, is constructed based on the sensed environmental cum vehicle data and the message is distributed to the passing vehicles. The work needs to be extended for highly dense scenarios.

Rokade et al. [23] developed an accident prediction model using multiple linear regression analysis for Bhopal city in India. This model considers an urban multilane road conditions along with past traffic data for accident prediction. This model works on the statistical data and not on real time data.

Saravanan et al. [24] developed an accident risk prediction model using fuzzy approach. The road, driver, and pedestrians are considered as risk factors for accident. This model can be used to analyze or investigate the road accident causation. This is a reactive model used for after investigation of a road accident.

It is learned from the literature that most of the existing models for accident prediction is based on either the vehicle parameters or the traffic parameters and most models are reactive and based on statistical data rather than real time data. Sometime there is a possibility of failure in the process of prediction because of not considering either of the above parameters. Hence in ESPM, both the parameters of vehicle status and traffic conditions are considered for emergency situation prediction process to ensure the performance.

## 4. Overview of ESPM

**Figure 3:**
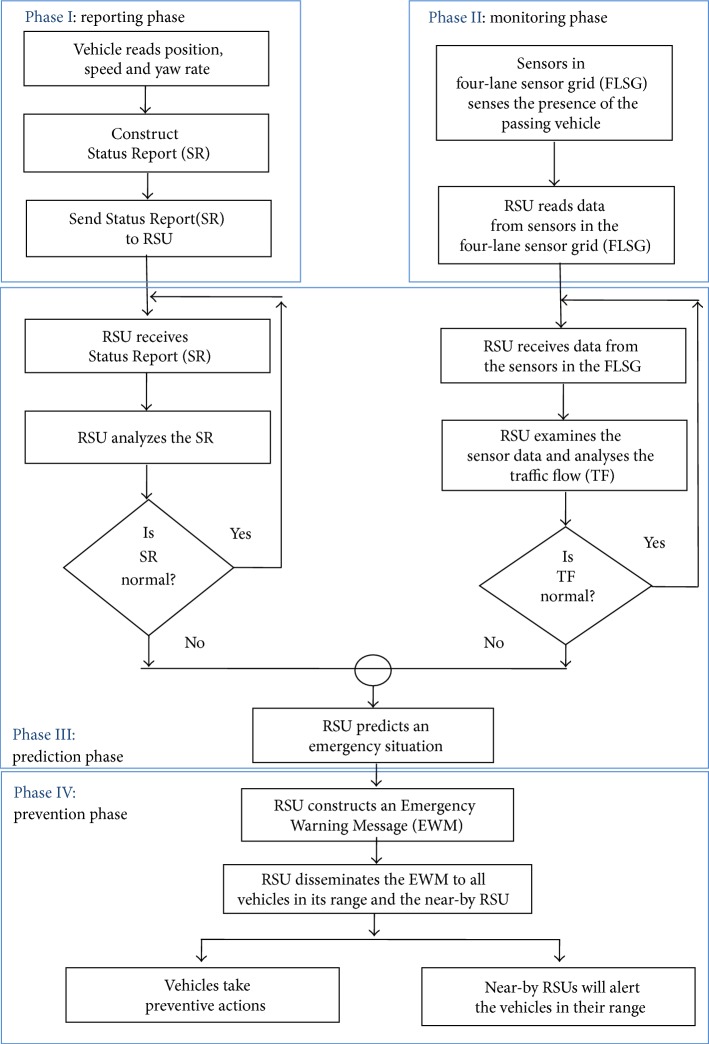
ESPM framework.

The proposed Emergency Situation Prediction Mechanism (ESPM) is a framework for traffic accident prediction and prevention. Overall structure of ESPM is shown in Figure 3. In ESPM, the vehicles, a four-lane highway, and road side infrastructure are participating in the processes of reporting, monitoring, prediction, and prevention phases.

ESPM performs prediction of emergency situation in three phases and prevention in the fourth phase. First, the participating vehicles will send Status Report (SR) to the near-by Road Side Unit (RSU). Second, RSU monitors the traffic flow in its range. Third, the RSU performs emergency situation prediction based on the SR and TF values and in fourth phase the RSU constructs and disseminates Emergency Warning Message (EWM) to all vehicles in its range and to the near-by RSUs. As a result the vehicles have a chance to take preventive action against accidents either by reducing the speed of the vehicle or taking an alternate route [22].

In this paper much attention is given to the first three phases and the fourth phase will be a future work.

## 5. Detailed Design

The ESPM framework is broadly divided into four phases, namely, reporting, monitoring, prediction, and prevention. Each phase is assigned a distinct task. The pseudo code of ESPM is presented below followed by a detailed discussion.

*Pseudocode of ESPM*

*Definitions*

 
*V*_ID_: vehicle ID;
 Pos: position of the vehicle;
 Sp: speed of the vehicle;
 Yr: yaw rate (i.e., rotation angle);
 SR: Status Report;
 FLSG: Four-Lane Sensor Grid;
 
*L*_*MN*_: *N*th block in *M*th lane of FLSG;
 
*S*_*MN*_: road sensor in *L*_*MN*_;
 Dm: decision matrix;
 GP_*MN*_: grid position in Dm (1 indicates presence of a vehicle);
 RSU: Road Side Unit;
 Periodic _Timer: set as 100 milliseconds;
 Dp: decision parameter;
 Td: traffic density.

*Reporting Phase*. See Algorithm 1.

*Monitoring Phase*. See Algorithm 2.

*Prediction Phase*

*Module 1*. See Algorithm 3.

*Module 2*. See Algorithm 4.

*Module 3*. See Algorithm 5.

### 5.1. Reporting Phase

It is assumed that each vehicle (a car) is equipped with (i) sensors for measuring speed and yaw rate (ii) Global Positioning System (GPS) for finding the location of the vehicle and (iii) transceivers for transmitting and receiving data [25].

**Figure 4:**
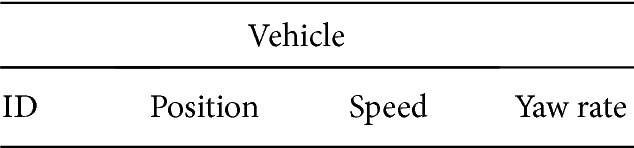
Components of Status Report (SR).

Once the vehicle enters into the range of the RSU, periodically (explained in Section 5.2.1), it has to send a Status Report (SR) to the RSU. The SR consists of the parameters such as* vehicle ID, position*,* speed*, and* yaw rate* of the vehicle as shown in Figure 4.

Where ID is used for the identification of the vehicle, position is the geographical position of the vehicle which is determined by the GPS device attached to the vehicle. Speed is determined by the speed sensor and yaw rate (i.e., rotation rate) is determined by yaw rate sensor. The location of the vehicle in a road lane can be calculated from the position and speed values of SR [20]. These vehicle ID, position, speed, and yaw rate are encapsulated in SR and reported to the RSU.

### 5.2. Monitoring Phase

In this phase, the RSU performs traffic flow monitoring based on the traffic flow data. The traffic flow data can be obtained with the help of sensors embedded in the four-lane express highway called Four-Lane Sensor Grid (FLSG).

#### 5.2.1. Four-Lane Sensor Grid

**Figure 5:**
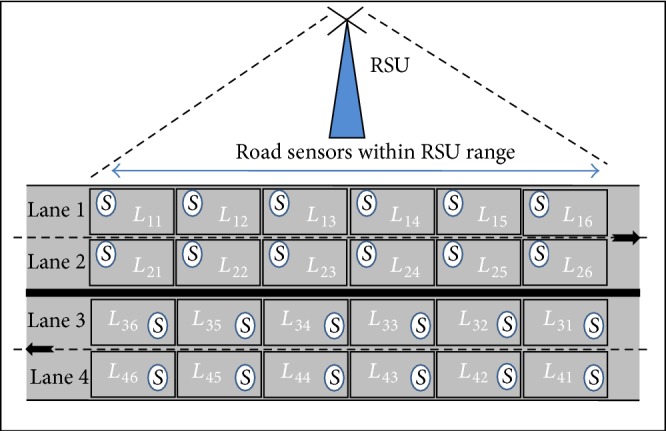
Organization of four-lane sensor grid (FLSG).

A Four-Lane Sensor Grid (FLSG) is a setup, where each lane is divided into blocks [26] of length 6 meters as shown in Figure 5 and each block is fixed with sensors (like Sensys sensors [27]). These sensors are capable of detecting the presence and passing of vehicles in the lane. This sensor data can be passed to an Access Point (AP) attached the RSU. A pair of sensors can be installed in the blocks of the same lane with same distance to measure the speed and length of the vehicles [27].

In FLSG, each block is denoted by *L*_*MN*_, where *M* and *N* are lane and block numbers, respectively, and *M* lies within 1 to 4 and *N* lies within 1 to 6. If a vehicle enters in block *L*_*M*1_ and travels along the blocks *L*_*M*2_, *L*_*M*3_,…, *L*_*MN*_ then the traffic flow might be normal. Otherwise, the traffic flow is considered to be abnormal.

In real time, lanes 1 and 2 are fixed with travel speeds 60 km/h and 90 km/h, respectively, in Indian four-lane express highway. Lanes 1 and 2 are symmetric to lanes 4 and 3, respectively. The width of each lane in FLSG is 3.6 meters and the width of the four lanes is 17 meters including the highway divider.

The total number of blocks in FLSG can be computed using

(1) $$\begin{array}{c} \overset{4}{\underset{M=1}{\sum }}\overset{6}{\underset{N=1}{\sum }}L_{MN}. \end{array}$$

The total number of Sensors in FLSG can be computed using

(2) $$\begin{array}{c} \overset{4}{\underset{M=1}{\sum }}\overset{6}{\underset{N=1}{\sum }}S_{MN}, \end{array}$$

where *M* and *N* are lane and block number, respectively.

The length of blocks in each lane is assumed to be 6 meters and the minimum and maximum speed limits of the lanes are fixed to be 60 km/h and 90 km/h, respectively, in simulation. Therefore the period for sending the SR is fixed as 0.24 seconds that is the minimum period computed for the above speed limits. (The maximum period is 0.36 seconds).

In this paper, the size of FLSG is considered as 24 blocks (i.e., *M* × *N*). The number of lanes can be increased dynamically up to 8 and the number of blocks can also be increased accordingly.

#### 5.2.2. Working of Monitoring Phase

The RSU periodically monitors the traffic flow in its range. In VANET, traffic flow can be predicted using past and present traffic flow data (i.e., the location dynamics of the vehicles). Traffic flow prediction is not possible only by considering past traffic data due to on road traffic accidents, off road events, and unavailability of traffic data in all links of a traffic network because most roads/links are not equipped with traffic sensors [28]. Due to this reason, in ESPM the four-lane highway is equipped with traffic sensors [26].

RSU is programmed in such a way that it receives data from Sensys sensors [27] placed in the blocks of each lane of the four-lane highway. With these sensor data, it would be possible for the RSU to identify the presence of a vehicle in a particular block of the FLSG. Similarly all the sensors will send the sensed traffic data to the RSU. That is, if the sensor senses the presence of a vehicle then it sets the grid position (GP) value as 1. Otherwise, the sensor sets the GP value as 0.

### 5.3. Prediction Phase

The prediction phase is instrumental for emergency prediction based on the SR and TF data received from reporting and monitoring phases, respectively. If either SR or TF values are abnormal then RSU predicts an emergency situation (i.e., the possibility of occurrence of an accident).

#### 5.3.1. Abnormality Based on the Status Report (SR)

Once the RSU receives the SR, it checks whether speed, position, and yaw rate are within the specified limits. If any of the following cases arise then RSU concludes that the SR is abnormal.

**Case 1 .**

If the position of the vehicle is the same in two continuous time periods then the vehicle is in halt state as represented by the following equation:

(3) $$\begin{array}{c} \text{Pos}\left(\;t_{i}\;\right)-\text{Pos}\left(\;t_{i-1}\;\right)=0. \end{array}$$

**Case 2 .**

If the difference in speed of the vehicle in two continuous time periods is greater than 30 km/h then the vehicle is with abnormal speed represented as

(4) Spti−Spti−130 km/h,Spti−1−Spti>30 km/h.

**Case 3 .**

If the vehicle's change in yaw rate exceeds 30 degrees then the vehicle has performed an abnormal lane change represented as

(5) Yrti−Yrti−130  degrees,Yrti−1−Yrti>30  degrees.

In (3), (4), and (5), Pos denote the position of the vehicle, Sp denotes the speed of the vehicle, and Yr denotes yaw rate of the vehicle and *t*_*i*_ is time.

#### 5.3.2. Abnormality Based on the Traffic Flow or Sensor Data (TF)

**Figure 6:**
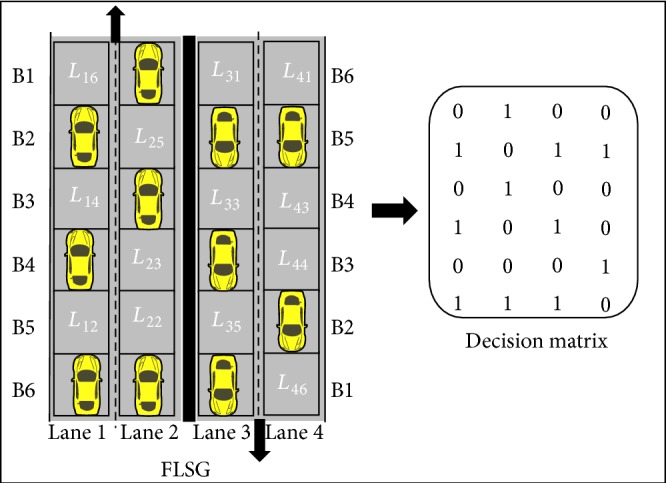
Transformation of four-lane sensor grid into decision matrix.

The RSU periodically receives the traffic data (i.e., GP value) through each sensor in FLSG; it transforms the traffic data into a decision matrix (DM) for traffic flow analysis as shown in Figure 6. The decision matrix is playing an important role in the prediction process.

If all the positions of DM are filled with 1s, then it means that the highway is filled with vehicles and the Traffic Density (TD) is high. If all the positions of DM are filled with 0s then the highway is free and TD is low. Three scenarios of abnormality are identified and explained below.

In FLSG, the sensors are fixed at the entry points of all the blocks. If a vehicle enters the block, immediately the sensor senses and reports to the RSU. The RSU records this by placing a “1” in the respective position of the decision matrix. The block size of FLSG is designed in such a way that, once a vehicle enters into a block means it automatically leaves the previous block (i.e., the sensor area in the previous block). Hence, if a vehicle is partially in two blocks the preceding sensor alone reports to the RSU about the presence of the vehicle.

**Scenario 1 (abnormal deceleration of the vehicle).**

Once the speed of the moving vehicle decreases to 0 Km/h (i.e., deceleration to 0 Km/h) (or) the position of the moving vehicle remains the same for two continuous time periods then it is concluded that the vehicles resides in the same position (or) stops travelling.

**Figure 7:**
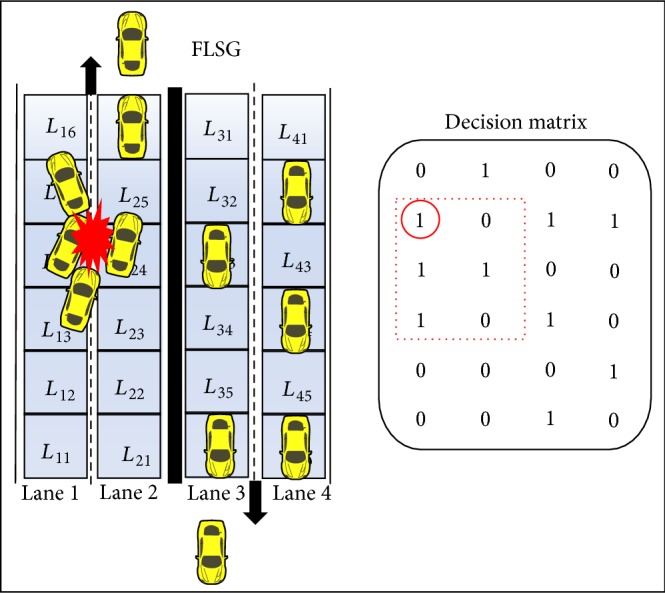
Scenario of abnormal deceleration with respective decision matrix.

If a set of vehicles travelling in a lane one after the other, sudden stoppage of a vehicle might cause rear end collision [9, 29] as shown in Figure 7. Major mechanical problems, driver illness, and improper driving might cause the vehicle to suddenly stop [24].

A vehicle in *L*_15_ of DM has abnormally decelerated; as a cause, vehicles in *L*_14_, *L*_13_, and *L*_24_ have collided (i.e., rear end collision) as shown in Figure 7. The computation of ESPM is based on the values of the decision matrix. With respect to DM, the vehicle representing the value of GP_15_ halts; hence the vehicles representing the values of GP_13_, GP_14_, and GP_24_ collides.

In DM, if a particular column is filled with continuous 1s then the probability of an emergency situation is much higher and the change in GP values of DM need to be closely monitored against abnormality.

**Scenario 2 (abnormal acceleration of the vehicle).**

If the moving vehicle suddenly accelerates its speed (or) the speed of the vehicle crosses the maximum lane speed limit then the vehicle might collide with the one before it (i.e., front end collision) [9]. This scenario might sometimes cause a series of front end collisions.

**Figure 8:**
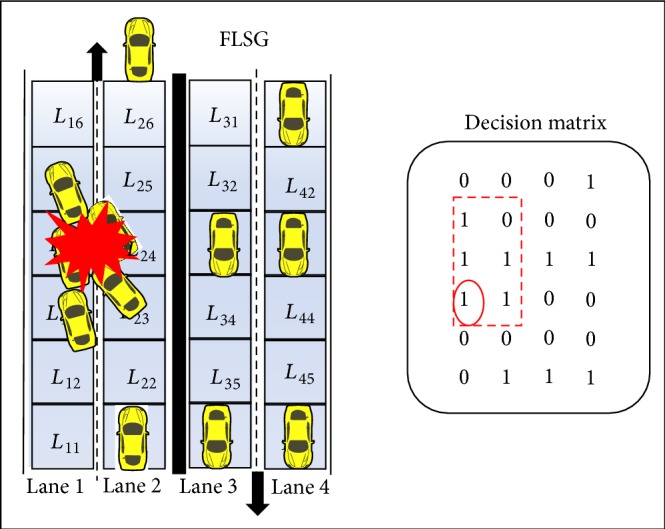
Scenario of abnormal acceleration with respective decision matrix.

In case vehicles are moving closely one after the other, if a following vehicle suddenly accelerates its speed abnormally then the following vehicle collides with the one in front (front end collision) and this might result in a chain of collisions as shown in Figure 8. The vehicle in *L*_13_ has accelerated abnormally which results in collision (front end collision) with the vehicles in *L*_14_, *L*_15_, *L*_23_, and *L*_24_. With respect to DM, the vehicle representing the value of GP_13_ has increased the speed abnormally. Hence the vehicles representing the values of GP_14_, GP_15_, GP_23_, and GP_24_ collide.

**Scenario 3 (abnormal lane change).**

The lane change the vehicle is normal only if the following condition is satisfied. Otherwise, the lane change is abnormal:

(6) $$\begin{array}{c} 1\geq V\left(\;M\;\right)\leq \frac{M_{\mathrm{Max}}}{2}, \end{array}$$

where *M* is the lane number, *V* is the vehicle, *M*_max⁡_ is the maximum lane number in the highway, and *V*(*M*) is vehicle's present lane.

The ESPM is designed for a four-lane express highway; the lane change is possible only between lane 1 and lane 2 in one direction and between lane 3 and lane 4 in the other direction (symmetric to the other direction). At par with expression (6), the vehicle can change its lane from lane 1 to lane 2 and vice versa. But a vehicle in lane 2 cannot perform lane change to either lane 3 or lane 4 because it causes head on accidents. Similarly a vehicle in lane 3 cannot perform lane change to either lane 1 or lane 2.

**Figure 9:**
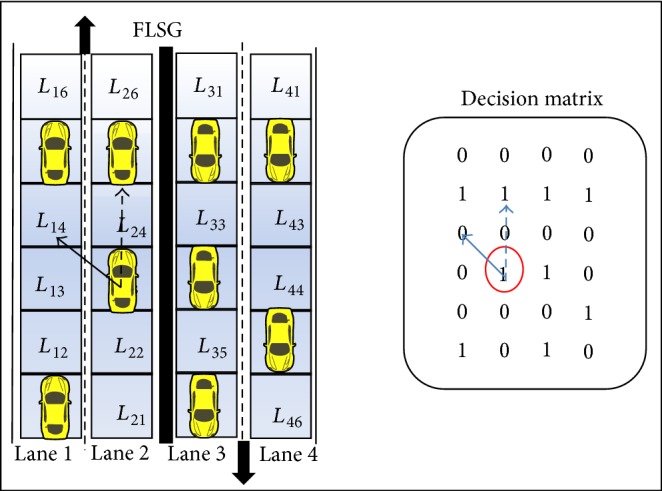
Abnormal inner to outer lane change with respective decision matrix.

Two types of lane changes are possible such as inner to outer (ITO) and outer to inner (OTI). Consider the present position of the vehicle in DM as GP_*MN*_; a smooth or normal ITO lane change is possible only if the position values (*M* − 1, *N* − 1), (*M* − 1, *N*), and (*M* − 1, *N* + 1) of DM are zero.  Figure 9 shows that a vehicle in *L*_23_ wants to change from lane 2 to lane 1. This lane change is normal only if GP_12_, GP_13_, and GP_14_ values of DM are zero.

**Figure 10:**
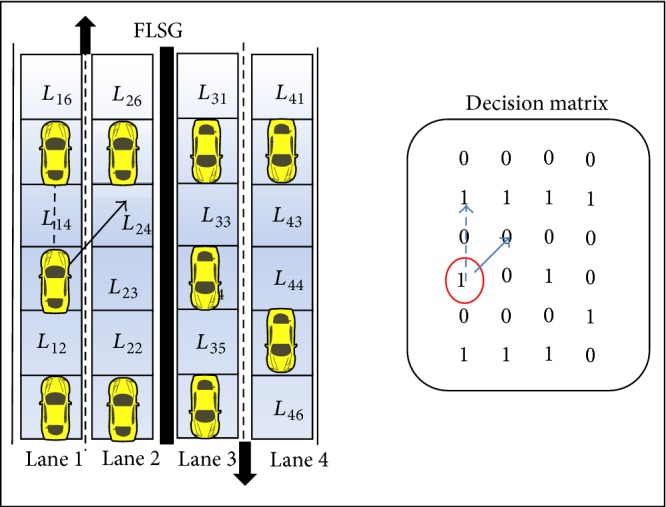
Abnormal outer to inner lane change with respective decision matrix.

Similarly, a smooth or normal OTI lane change is possible only if the position values (*M* + 1, *N* − 1), (*M* + 1, *N*), and (*M* + 1, *N* + 1) of DM are zero.  Figure 10 shows that a vehicle in *L*_13_ wants to change lane from lane 1 to lane 2. This lane change is normal only if GP_22_, GP_23_, and GP_24_ values of DM are zero.

Otherwise, both the lane changes would be abnormal and this leads to an emergency situation.

If any of the above three scenarios arise then it could be concluded that there is a possibility of occurrence of an accident and the prediction is successful (i.e., an emergency situation is predicted).

### 5.4. Prevention Phase

This prevention phase is purely dependent on the prediction phase. Once the emergency situation is predicted by the prediction phase, then the RSU constructs an Emergency Warning Message (EWM). This EWM is then broadcasted by the RSU to all the vehicles and near-by RSUs in order to alert them about the possibility of occurrence of an accident. The near-by RSUs will broadcast this emergency message to the vehicles in their respective ranges. Upon reception of EWM, the vehicles might prevent themselves from accidents either by stopping or taking alternate lanes/routes.

## 6. Simulation Setup

A mobility scenario for highway traffic in Indian four-lane express highway has been developed using Freeway Mobility (FM) model. The FM model generates common scenarios such as stopping, lane changing, and overtaking in highways. The FM model injects vehicles in each lane at a specific traffic rate. As specified in Figure 2, vehicles of lane 1 and lane 2 will move in one direction against the vehicles of lane 3 and lane 4 in the opposite direction. During simulation the vehicles are placed randomly in four lanes. The speed of each vehicle in FM model [30] is based on the following:

(7) $$\begin{array}{c} \text{Sp}\left(\;t_{i+1}\;\right)=\text{Sp}\left(\;t_{i}\;\right)+\text{random}\left(\right)\times A\left(\;t_{i}\;\right). \end{array}$$

The FM model does not allows lane change; hence (7) has been changed with yaw rate and modified as

(8) $$\begin{array}{c} \text{Sp}\left(\;t_{i+1}\;\right)=\left[\;\text{Sp}\left(\;t_{i}\;\right)+\text{random}\left(\right)\times A\left(\;t_{i}\;\right)\;\right]\times \text{ROT}, \end{array}$$

where Sp(*t*_*i*_) is the speed of the vehicle at time “*t*,” random() provides a random value in between [−1,1] used for both acceleration and deceleration of vehicles, and *A*(*t*_*i*_) is the vehicle's acceleration at time “*t*” and ROT is Radius Of Turn [31] and can be expressed as

(9) $$\begin{array}{c} \text{ROT}=\frac{\text{Sp}\left(t_{i}\right)}{\text{Yr}}, \end{array}$$

where Yr is yaw rate at maximum and expressed as

(10) $$\begin{array}{c} \text{Yr}=\frac{\left(2\text{Sp}\left(t_{i}\right)\right)\times \sin\theta }{d}, \end{array}$$

where *d* is the distance of travel [32].

Network Simulator (ns 2.34) is used for simulation. The mobility traces of the scenarios (i) sudden stoppage (deceleration), (ii) sudden increase in speed (acceleration), and (iii) abnormal lane change of vehicles are developed using the FM model.

The simulation is performed on two layers of nodes. To simulate the fixed RSU and FLSG sensors nodes one (fixed) layer of nodes are used. The second layer of nodes is the moving vehicles whose mobility traces are opted from the FM model. The main simulation parameters are listed in Table 1. As two lanes in four-lane highway are symmetric to the other two lanes in the opposite direction, only two lanes are considered for simulation.

The moving vehicles will send SR to the fixed RSU nodes once in every 100 milliseconds (lesser that the required 240 milliseconds as explained in Section 5.2.1). Similarly the fixed sensor nodes in FLSG will send the data about presence of the vehicle in its coverage.

Based on both the SR and sensor data, the RSU node will analyze the abnormal behavior of vehicles and arrive at a conclusion about the possibility of occurrence of an accident (i.e., prediction). The vehicle might behave abnormally in any of the scenarios as mentioned in the previous Section 5.

## 7. Results 

The results demonstrate the efficiency of the ESPM framework for emergency situation prediction. Prediction probability and vehicle density are selected as performance metrics for evaluating the performance of ESPM.

In order to demonstrate the accuracy of the ESPM approach the following three scenarios were simulated.

**Scenario 1 (abnormal deceleration of the vehicle (sudden stoppage of a moving vehicle)).**

In this scenario, initially a moving vehicle (mobile node) in a lane has been forced to stop by making its speed reach 0 km/h instantly and the reaction in the system has been monitored. After receiving the SR of the respective vehicle, the RSU node has reported an abnormality within the specified time period and a successful prediction has been achieved.

**Figure 11:**
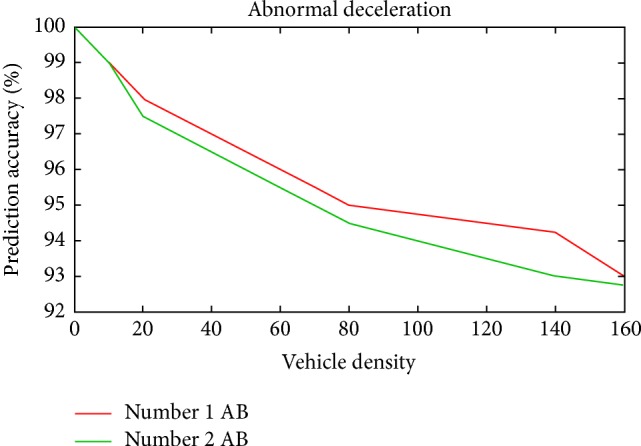
Prediction accuracy versus vehicle density for Scenario 1.

The simulation was repeated for 10 runs by gradually increasing the vehicle density and randomly vehicles in the two lanes were made to stop suddenly. The reaction of both the vehicles and RSU has been monitored for accuracy and performance of ESPM. In Figure 11, the simulation results, prediction accuracy (PA) against vehicle density (VD) for one and two abnormal behaviors (AB) are above 92 percent.

**Scenario 2 (abnormal acceleration of the vehicle (sudden increase in speed)).**

In this scenario, among the moving vehicles (mobile nodes) in two lanes a vehicle was forced to increase its speed to cross the maximum limit of 90 km/h and the systems' reaction has been monitored. The RSU node has reported an abnormality within the specified time period after reception of SR from the respective vehicle. Similarly the speed of the vehicles in two lanes was increased and the reaction of the RSU node has been monitored.

**Figure 12:**
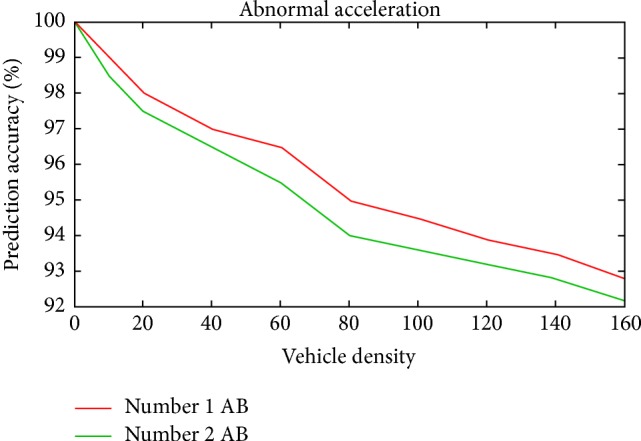
Prediction accuracy versus vehicle density for Scenario 2.

This simulation was repeated for 10 runs by gradually increasing the vehicle density (i.e., number of vehicles). The vehicle behavior and reaction of RSU was closely monitored for assessment of performance and the same is shown in Figure 12.

The simulation result shows that the prediction accuracy is close and above 92 percent both abnormal behaviors (AB). Comparing both Figures 11 and 12, prediction accuracy in Scenario 2 for two abnormal behaviors is slightly decreasing when comparing to Scenario 1.

**Scenario 3 (abnormal lane change (inner to outer and outer to inner)).**

During simulation in this scenario, vehicles were made to change their lanes from inner to outer and outer to inner in different runs.

(a) A vehicle was allowed to perform a normal inner to outer (ITO) lane change and forced to perform an abnormal inner to outer lane change as explained in Section 5.

(b) Similar to the previous case a vehicle was allowed to perform a normal outer to inner (OTI) lane change and forced to perform an abnormal outer to inner lane change as explained in Section 5. Then the reaction of both the sensor nodes and RSU has been monitored. After receiving data from the sensor nodes, the RSU computed normal and abnormal lane changes within the specified time period. In the case of abnormality, RSU records the abnormality to report successful prediction of an emergency situation.

**Figure 13:**
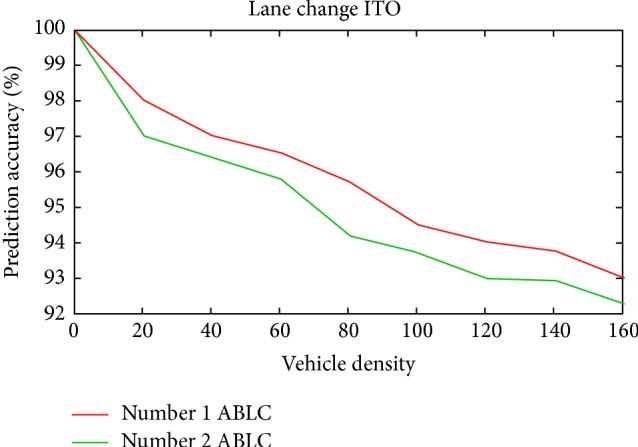
Prediction accuracy versus vehicle density for Scenario 3(a).

**Figure 14:**
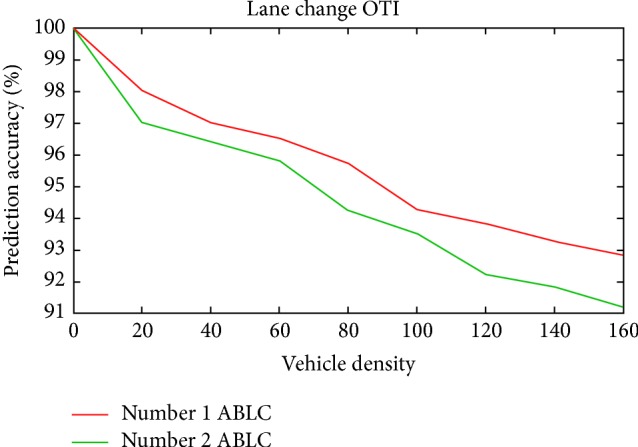
Prediction accuracy versus vehicle density for Scenario 3(b).

The prediction performance of ESPM in this scenario is promising and this is shown in Figures 13 and 14 for inner to outer (ITO) and outer to inner (OTI) lane changes, respectively. During simulation one and two abnormal lane changes (ABLC) were induced to measure the accuracy of prediction.

Figure 13 shows that the PA in Scenario 3(a) (ITO) is almost close to 91 percent for both one and two abnormal behaviors, respectively, whereas the prediction accuracy of Scenario 3(b) (OTI) is decreasing when comparing the previous one as in Figure 14.

The speed limit of inner lane is greater than the speed limit of outer lane. This leads to the increase of complexity in OTI lane change when comparing to ITO lane change. Due to this reason the prediction accuracy of OTI lane change might decrease.

A vehicle attempting for OTI lane change must increase its speed from 60 km/h range to 90 km/h range in order to meet the speed of vehicles in the inner lane. At par with (8) the speed of the vehicle performing lane change should cope up with the yaw rate and lane speed limit.

In all the three scenarios, the simulation results show that the successful prediction achieved within 0.2 seconds. This is much better than the statistical requirements of 0.5 seconds [7]. It is further noticed that if the vehicle density is less than the prediction accuracy is high but when the vehicle density increases the prediction accuracy decreases to close and above 92 percent. The average vehicle density is around 60 vehicles/second/lane in Indian four-lane express highways; hence this level of accuracy is acceptable to these road conditions.

## 8. Conclusion and Future Work

In this paper, an Emergency Situation Prediction Mechanism (ESPM) is proposed to predict the possibility of occurrence of an accident in Indian four-lane express highway. The primary objective of ESPM is to predict an emergency situation in advance, thereby preventing accidents and reducing the death toll. ESPM is used to perform prediction of an emergency situation in four phases. The first three phases (reporting, monitoring and prediction phases) are used for prediction and the fourth phase is used for prevention. In ESPM, the prediction accuracy is computed against vehicle density for three different scenarios. In all the three scenarios, it is noticed that there is a small gap between the analytical evaluation and simulation results. The results show that the performance of ESPM is promisingly increased towards prediction. Prediction accuracy of ESPM against vehicle density is almost above 92 percent.

Implementation of ESPM for Indian four-lane express highways is expensive because of the development and deployment of infrastructures such as FLSG and RSU even though most of the present day vehicles are automated vehicles. But in near future ESPM has a high scope of implementation for improving ITS.

In future, attempts will be made to improve the performance of ESPM. Based on prediction of emergency situation the prevention of accidents would be done by disseminating distress beacons (i.e., Emergency Warning Message) to alert all vehicles in the range of the RSU and the vehicles in the range of the near-by RSUs to prevent from accidents.

## Figures and Tables

**Algorithm 1:**
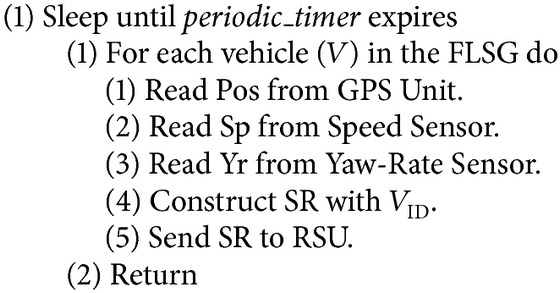


**Algorithm 2:**
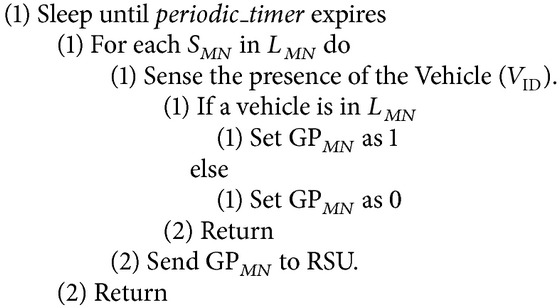


**Algorithm 3:**
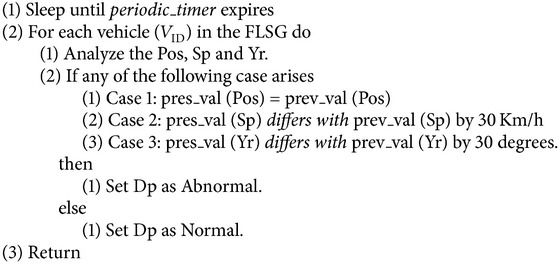


**Algorithm 4:**
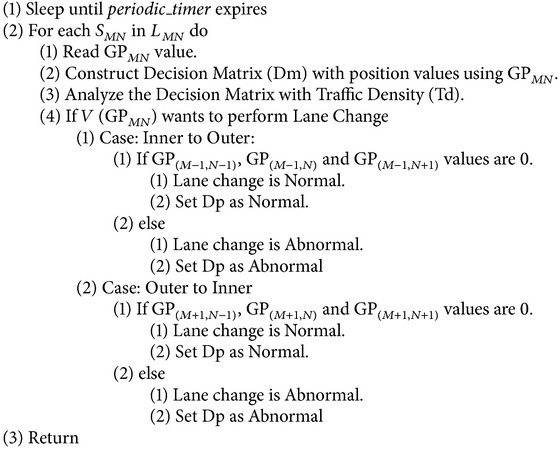


**Algorithm 5:**
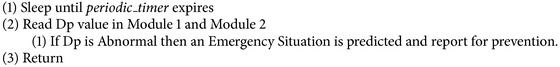


**Table 1:** Simulation parameters.

Parameter	Value
Highway scenario dimension	1000 m, 2 lanes, and 1 direction
Simulation time	300 s
Assumed vehicle length	5 m
Vehicle speed limits	60–90 Km/h
Average speed	70 Km/h
Vehicle density	50–70 vehicles per 500 m
Traffic rate/vehicle injection rate	(1/75, 1/60, 1/45, 1/15, 1/5) vehicles/sec/lane
Vehicle transmission range	500 m
Sensing radius of sensors	6 m
Number of vehicles	150
Number of road sensors	200
Number of lanes	2 lanes
Distance between two sensors in a lane	6 m
Distance between two sensors in two neighboring lanes	3.6 m
Period of message exchange	0.10 second (i.e., 100 milliseconds)
